# 

**Published:** 1841

**Authors:** 


VOL I.—NO. IX & X.

EDITED BY

CHAPIN A. HARRIS, M. D.
BALTIMORE.

ELEAZAR PARMLY, M. D.
NEW-YORK.

PUBLISHING COMMITTEE,
E. PARMLY, M.D. | E. BAKER, M. D.
S. BROWN, M. D.

NEW YORK,

GEORGE ADLARD, 168 BROADWAY,

BALTIMORE,

ARMSTRONG & BERRY.

PRICE---THREE DOLLARS PER ANNUM.

J. A. FRAETAS, PRINTER.

